# Field-deployable molecular diagnostic platform for arbovirus detection in *Aedes aegypti*

**DOI:** 10.1186/s13071-020-04357-y

**Published:** 2020-09-24

**Authors:** Natalie Rutkowski, Yuemei Dong, George Dimopoulos

**Affiliations:** grid.21107.35 0000 0001 2171 9311 W. Harry Feinstone Department of Molecular Microbiology and Immunology, Bloomberg School of Public Health, Johns Hopkins University, Baltimore, MD USA

**Keywords:** *Aedes aegypti*, Zika virus, Dengue virus, Diagnostics, qPCR

## Abstract

**Background:**

Surveillance of mosquito infection status is critical for planning and deployment of proper mosquito control initiatives. Point-of-care (POC) detection assays are necessary for monitoring the infection prevalence and geographical range of viruses in mosquito vector populations. We therefore assessed the novel real-time PCR (qPCR) bCUBE (Hyris, London, UK) molecular diagnostic system as a tool for virus detection.

**Methods:**

*Aedes aegypti Rps*17 was used to validate and determine correlation coefficient for the novel bCUBE qPCR system to a laboratory standard StepOnePlus real-time PCR system (Applied Biosystems, Waltham, MA, USA). Experimentally infected *Ae. aegypti* were quantified for Zika (ZIKV) and dengue virus serotype 2 (DENV2) viral genomic RNA. Infection prevalence was compared to plaque assay.

**Results:**

We developed and validated a novel qPCR system for the detection of ZIKV and DENV2 using the real-time qPCR system bCUBE. With bCUBE-based qRT-PCR, viral genomic RNA could be detected in individually infected *Ae. aegypti* mosquitoes and in pools of 5, 10 or 15 mosquitoes.

**Conclusions:**

The portable qPCR bCUBE diagnostic system is capable of detecting Zika and dengue virus in mosquitoes and therefore has potential as a practical field-deployable diagnostic test for vector-borne disease surveillance programmes.
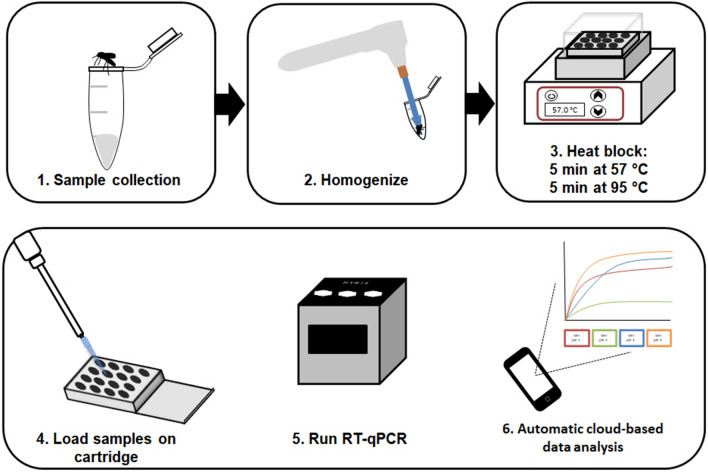

## Background

Arthropod-borne diseases threaten over two-thirds of the global population and are exhibiting an ongoing expansion of their geographical range and prevalence as a result of climate change, urbanization and globalization [1–4]. According to the World Health Organization, dengue virus infection has increased 30-fold over the past 50 years and now affects nearly 100 million people, primarily in the Americas and Asia [5, 6]. The outbreaks of Zika in 2015 and 2016 have also led to widespread concern because of the virus ability to cause newborn malformations [7, 8].

Dengue and Zika viruses are members of the genus *Flavivirus* that are primarily transmitted by the *Aedes* mosquitoes [7, 9, 10]. Other members of the genus *Flavivirus* are also recognized as vector-borne pathogens of public health significance, including West Nile virus (WNV), yellow fever virus (YFV), Japanese encephalitis virus (JEV) and chikungunya virus (CHIKV). These viruses cause similar flu-like symptoms with the potential to progress to neuroinvasive outcomes. *Aedes* mosquitoes, with their aggressive blood-feeding behavior, have allowed for efficient human-mosquito transmission of these arboviruses [3, 11]. The geographical presence of this mosquito vector has dramatically increased in the last few decades, leading to an expanded transmission of these arboviruses [12–14]. The lack of vaccines and treatment against these arboviruses highlights the importance of mosquito control and surveillance strategies [15]. Current and future mosquito-targeted control strategies have, and will have, a significant epidemiological impact but also require robust mosquito and pathogen surveillance [15]. Surveillance of geographical distribution of the vector mosquitoes and the pathogens they carry is an essential component of disease prevention and control.

Accurate, rapid, and cost-effective surveillance of vector-borne pathogens is critical for monitoring infection prevalence and thereby mitigating transmission risk. Historically, standard cell culture assays were used for detecting live virus; however, more efficient and faster ways to detect virus in field mosquitoes was necessary [16]. Methods currently used for arboviral detection include viral culture, antibody detection, antigen detection, and RNA detection using quantitative real-time RT-PCR (qPCR) [17, 18]. Currently, the fluorescent-labeled oligonucleotide probe-based qPCR method, such as TaqMan™ (Applied Biosystems, Thermo Fisher Scientific, Waltham, MA, USA), is the gold standard because of its ability to amplify sequence-specific molecular markers [19]. Many of these assays are laborious and laboratory-based and require expensive and bulky instruments, making them incompatible with low-resource regions [20]. Recently, several novel, advanced point-of-care (POC) diagnostic measures have been developed for detecting mosquito-borne viruses in the field, including honey-baited nucleic acid preservation cards [21, 22], loop-mediated isothermal amplification (LAMP) [23], biosensors [24], and adaptations of near-infrared spectrometry techniques [25]. However, these techniques have documented limitations, including cross-reactivity with other flaviviruses or requirements for training on specialized equipment, making the adoption of these new diagnostic tools difficult [26, 27]. Although the global burden of emerging outbreaks of Zika and dengue is clearly recognized, there is a gap in resources for endemic countries and regions, which are consistently plagued by a lack of equipment and adequate resources to consistently monitor the prevalence and range of infected mosquitoes [5, 28, 29].

Disease surveillance and integrated vector control are essential for curbing disease transmission. Nucleic acid-based testing to detect viral genomic RNA allows for specific and sensitive virus monitoring in mosquito surveillance programmes [20]. Several conventional and more recent real-time PCR-based assays have been established for mosquitoes and their vectored pathogens, and the ready availability of genome sequences for both vectors and pathogens can support the identification of additional PCR-compatible molecular markers [30–32].

In the present study, we evaluated a novel and portable real-time PCR platform, bCUBE (Hyris Ltd, London, UK), as a PCR-based arboviral detection method with potential for field deployability. bCUBE makes possible the genetic testing of biological samples in any setting, at any time, with real-time access to results on its dedicated cloud-based software platform. This technology is a portable device (10 × 10 × 12 cm), similar in size to a Bluetooth speaker that fits in one hand, capable of performing thermocycling reactions such as real-time PCR, as well as loop-mediated isothermal amplification (LAMP). The bCUBE is already being used for biological analysis in several fields, including agricultural pest control. The device can be operated from a laptop, tablet, or smart phone through an easy-to-use gateway and generates centralized data analysis immediately after a reaction. The cloud-based software can be calibrated to distinguish between positive and negative samples in a single reaction with predetermined conditions that have been established ahead of time in the laboratory. This feature allows the bCUBE to be operated by individuals lacking in-depth training in qPCR assays and data analysis skills. We have now explored the use of bCUBE technology for detection of both dengue and Zika virus in *Aedes aegypti,* optimizing and standardizing the sample preparation method to be used with a commercially available one-step qRT-PCR assay kit. Finally, we have developed a bCUBE-compatible qPCR diagnostic assay for the surveillance of arboviral pathogens in *Aedes* mosquitoes.

## Methods

### Mosquito rearing and mosquito infections

*Aedes aegypti* Liverpool strain LVP-IB12 and *w*AlbB-infected *Ae. aegypti* (Waco strain) WB1 mosquitoes [33] were maintained on 10% sucrose solution under standard insectary conditions at 27 ± 0.5 °C and 75–80% humidity with a 12:12 h light:dark photoperiod. Mosquitoes were reared using a standard rearing protocol established at John Hopkins Malaria Research Institute Insectary Core Facility as described before [34] and colonies were maintained on Swiss Webster mice (Charles River Laboratories International Inc., Wilmington, MA, USA).

*Aedes aegypti* mosquitoes were experimentally infected with ZIKV (*n* = 160) and DENV2 (*n* = 160) through an artificial membrane glass feeder containing anonymous human blood (O +) and heat-inactivated human serum (InterstateBlood Bank Inc., Memphis, TN, USA) supplemented with the corresponding virus from cell line cultures (see below). Samples from the mosquitoes were evaluated for infection status with the bCUBE platform at 7- and 14-days post-infectious blood meal (PIBM) [35].

Infected mosquitoes were double-caged and incubated in a reach-in incubator under conditions similar to those in the standard insectary chamber described above.

### Cell culture and virus propagation

*Aedes albopictus* C6/36 cells (ATCC CRL-1660) were cultured and viral stocks were prepared as previously described [36]. In brief, C6/36 cells were cultured in MEM medium (Gibco, Thermo Fisher Scientific, Waltham, MA, USA) supplemented with 10% heat-inactivated fetal bovine serum (FBS), 1% penicillin–streptomycin and 1% non-essential amino acids and maintained in a tissue culture incubator at 32 °C and 5% CO_2_. Baby hamster kidney strain 21 (BHK-21, ATCC CCL-10) cells were maintained at 37 °C and 5% CO_2_ in the DMEM medium (Gibco, Thermo Fisher Scientific) supplemented with 10% fetal bovine serum (FBS), 1% penicillin–streptomycin and 5 µg/ml Plasmocin (InvivoGen, San Diego, CA, USA). DENV serotype 2 New Guinea C strain (DENV2) and ZIKV strain FSS 13025 (ZIKV) were used in the indicated experiments. For viral stock preparation, C6/36 cells grown to 80% confluence were infected with ZIKV and DENV2 at a multiplicity of infection (MOI) of 10 and incubated at 32 °C and 5% CO_2_ for 5 days or 6 days for DENV2 or ZIKV, respectively. Virus was harvested by three freeze–thaw cycles using dry ice and a water bath (37 °C), then centrifuged at 2000× *rpm* for 10 min at 4 °C. The supernatant from this cell lysis was mixed with the original cell culture supernatant to yield the final viral stock. Viral stocks were aliquoted and stored at − 80 °C for long-term storage. Viral stock titration was done by plaque assay.

### Viral titration by plaque assay

The titers of ZIKV and DENV2 in the original viral stocks and in the infected mosquitoes were determined by plaque assays in BHK-21 cells as described in [37] with modifications. Viral titers used to infect mosquitoes were 1.0 × 10^6–8^ for DENV2 and 1.0 × 10^6–9^ for ZIKV. Three experiments were performed. Whole mosquito or mosquito tissue samples were collected at 7- and 14-days PIBM in 150 µl of complete DMEM medium with glass beads. Tissue samples were homogenized with a Bullet Blender (Next Advance, Troy, NY, USA) and serially diluted with DMEM complete medium. One or two days before plaque assay, the BHK-21 cells were split at a 1:10 dilution and grown on 24-well plates to 80% confluency. Serially diluted mosquito or viral samples (100 µl each) were added to the BHK-21 cells, followed by incubation at room temperature for 15 min on a rocking shaker (VWR International LLC, Radnor, PA, USA) and subsequent incubation at 37 °C with 5% CO_2_ in a cell incubator (Thermo Fisher Scientific) for another 45 min. The 24-well plates with infected BHK-21 cells were overlaid with 1 ml of 0.8% methylcellulose in complete DMEM medium with 2% FBS and incubated for 5 to 6 days in the cell culture incubator (37 °C and 5% CO_2_). Plaques were fixed and developed with staining reagent (1% crystal violet in 1:1 methanol/acetone solution) at room temperature for approximately 30 min. Plates were rinsed with DI water and air-dried and plaques were counted and multiplied by the dilution factor to calculate the plaque forming units (PFUs) per sample.

### Primer design for real-time quantitative RT-PCR (qPCR)

The ZIKV envelope (E) protein was chosen as the target for ZIKV detection and primers were developed based on previously established sequences [38]. Dengue virus serotype 2 (DENV2) qPCR was performed using previously developed protocols that employ primers detecting serotype 2 in the 3′-UTR region (Table 1) [39]. The primers were modified and optimized for bCUBE qPCR as listed in Table 1. Briefly, the ZIKV forward primer targets the E protein sequences at 65 base pairs (bp) upstream of the forward primer developed by Lanciotti et al. [38]. The ZIKV reverse primer was shifted downstream 4 bp and one additional nucleotide was added at the end. For DENV2 primer pairs, the forward primer was redesigned due to the non-specificity of the published primer [39] while the reverse primer was kept the same as published in [39]. NCBI BLAST was used to assess the specificity of these new designed primers. Previously established primers for *w*AlbB detection were used for *Wolbachia-*infected *Ae. aegypti* (Table 1) [40].

**Table 1:** Primer pairs for viral detection and cross-reactivity panel for real-time qPCR using the bCUBE

Primer	Sequence (5′–3′)	Nucleotide position	Amplicon size (bp)		Accession number	References
ZIKV-forward	AGCAACATGGCGGAGGTAAG	1128–1147	145	FSS13025		This study; [34]^a^
ZIKV-reverse	CTGTCCACTAACGTTCTTTTGCAGA	1249–1273				
DENV2-forward	TCCCTTCCAAATCGCAGCAACAATG	10,517–10,541	168	NC_001474.2		This study; [35]^b^
DENV2-reverse	CGTTCTGTGCCTGGAATGATG	10,665–10,685				
*w*AlbB-forward	CCTTACCTCCTGCACAACAA	213,522–213,541	110	CP031221.1		[36]
*w*AlbB-reverse	GGATTGTCCAGTGGCCTTA	213,394–213,412				
WNV-forward	TTGTGTTGGCTCTCTTGGCGTTCTT	233–257	408	AF196835		[38]
WNV-reverse	CAGCCGACAGCACTGGACATTCATA	640–616				
JEV-forward	GGCAGAAAGCAAAACAAAAGA	390–410	367	AF080251		[39]
JEV-reverse	CGGATCTCCTGCTTCGCTTGG	736–756				
YFV-forward	CACGGCATGGTTCCTTCCA	5656–5674	71	MN708497		[37]
YFV-reverse	ACTCTTTCCAGCCTTACGCAAA	5707–5728				
CHIKV-forward	TACAGGGCTCATACCGCATC	10,357–10,376	154	NC_004162		[40]
CHIKV-reverse	AAAGGTGTCCAGGCTGAAGA	10,492–10,511				

^a^ZIKV primers were modified and optimized for qRT-PCR

^b^DENV2 primers were modified and optimized for qRT-PCR

*Notes*: The cross-reactivity panel primer sequences were included to confirm viral RNA obtained from BEI Resources. Four viruses were included in this study: West Nile virus (WNV), Japanese encephalitis virus (JEV), yellow fever virus (YFV) and chikungunya virus (CHIKV). Primer sequence, nucleotide position and amplicon size are listed

### Cross-reactivity panel of mosquito-borne viruses

The frequently co-circulating arboviral RNA samples included in the cross-reactivity panel were chikungunya virus (CHIKV, H20235 ST MARTIN 2013), Japanese encephalitis virus (JEV, India R53567), West Nile virus (WNV CO 1862) and yellow fever virus (YFV 17D), obtained from BEI Resources (Table 2). RNA concentrations were measured using a NanoDrop Spectrophotometer (Thermo Fisher Scientific) and viral RNA samples for each specific virus were confirmed using previously established primers and qRT-PCR (Table 1) [41–44].

**Table 2:** Evaluation of the specificity of bCUBE-based qRT-PCR for DENV2 and ZIKV detection

Family	Genus	Species	Strain	BEI No	RNA concentration (ng/µl)	No. of positives for ZIKV assay	No. of positives for DENV2 assay
*Flaviviridae*	*Flavivirus*	Japanese encephalitis virus	India	NR-9592	0.1013	0/3	0/3
		West Nile virus	CO 1862	NR-50434	0.10	0/3	0/3
		Yellow fever virus	17D	NR-2869	0.120	0/3	0/3
*Togaviridae*	*Alphavirus*	Chikungunya virus	St Martin 2013	NR-50130	0.167	0/3	0/3

*Notes*: A cross-reactivity panel of frequently co-circulating viruses was used to evaluate the specificity of bCUBE-based qRT-PCR for DENV2 and ZIKV. Arboviral RNA samples were obtained from BEI Resources. RNA concentration (ng/µl) and number of positively detected samples from the ZIKV and DENV2 assay are listed

### Total RNA preparation for DENV2- and ZIKV-infected whole mosquitoes or tissues

Total RNA extraction was performed using a squash buffer (10 mM Tris pH 8.2, 1 mM EDTA, 50 mM NaCl) [45]. Individual whole mosquito or tissue samples were collected at two time points PIBM. Abdomens with midguts (ABD) were collected at 7 days and heads with thoraces (HT) were collected at 14 days to correspond to viral dissemination in the mosquito. All six legs (L), corresponding to identical heads with thoraces, were collected at 14 days PIBM. ABD and HT tissues or L samples were collected in 50 µl or 20 µl squash buffer, respectively, and stored at -80 °C until extracted. Proteinase K (Qiagen) was added at 1/8 volume to give a final concentration of 15 mg/ml and homogenized with a cordless Pellet Pestle Motor (Kimble Kontes, NJ, USA) for 40–60 s. Samples were incubated at 57 °C for 5 min, followed by 95 °C for 5 min for enzyme deactivation. The supernatant from this crude RNA extraction was used immediately for qPCR or stored at − 80 °C until use [45].

### Pooled DENV2- and ZIKV-infected mosquito samples

Preliminary pooled sample experiments involved a total of 300 *Ae. aegypti*: 276 uninfected, 12 infected with DENV2 and 12 infected with ZIKV. First, 12 mosquitoes were infected with ZIKV and 12 with DENV2. Following confirmation of infection by bCUBE RT-qPCR of individual infected mosquitoes, each was placed into a pool of uninfected mosquitoes. Four different pools were used to measure the sensitivity of the infection detection for pools of 5, 10, 15 and 20. The squash buffer volumes used were 250, 450, 700 and 950 µl, respectively. Samples were processed as described above.

### gDNA preparation of *Wolbachia*-infected mosquitoes

Total gDNA from *Wolbachia-*infected total mosquitoes was prepared as described previously and above [45]. These crude gDNA extractions were immediately used for qPCR or stored at − 80 °C until use.

### cDNA preparation

For correlation studies, *Ae. aegypti* RNA was extracted using TRIzol Reagent (Invitrogen, Thermo Fisher Scientific, Waltham, MA, USA) according to the manufacturer’s instructions. RNA concentration was measured by NanoDrop Spectrophotometer (Thermo Fisher Scientific). Approximately 1–2 µg total RNA was used for cDNA synthesis. cDNA was synthesized using M-MLV Reverse Transcriptase Kit (Promega, Madison, WI, USA) according to the manufacturer’s instructions. The resulting cDNA was serially diluted and 1 µl of cDNA was used as template for qPCR analysis.

### Laboratory standard real-time PCR

A laboratory standard StepOnePlus Real-Time PCR System (Applied Biosystems, Thermo Fisher Scientific) was used for comparative analysis to validate portable bCUBE qPCR system. The *Ae. aegypti* housekeeping *Rps*17 (ribosomal protein S17 gene) primers (Forward: 5′-CAC TCC CAG GTC CGT GGT AT-3′; Reverse: 5′-GGA CAC TTC CGG CAC GTA GT-3′) were used with *Ae*. *aegypti* crude gDNA extract and SYBR Green PCR Master Mix (Applied Biosystems, Thermo Fisher Scientific) as described in [37]. Mosquitoes were collected individually in 50 µl of squash buffer and crude gDNA was extracted.

### Hyris bCUBE real-time RT-PCR

The portable qPCR machine Hyris bCUBE 2.0 thermocycler (Hyris, London, UK) and the 16-well cartridges were kindly provided by Hyris Inc. The Hyris data analysis platform was used for this study. GoTaq 1-Step RT-qPCR Kit (Promega, Madison, WI, USA) was used at a final volume of 20 µl with 0.4 µl of kit reverse transcriptase enzyme, 10 µl of RT-PCR buffer, 0.3 µl of 10 µM of forward and reverse primer, 9 µl of DNA/RNA-free water and 1 µl of crude RNA or gDNA sample. Each sample was performed in technical duplicate. Each cartridge run included one negative and one positive control. The following thermocycling settings were used: reverse transcription for 15 min at 37 °C, heat-inactivation of reverse transcriptase at 95 °C for 10 min, followed by 30 PCR cycles at 95 °C for 10 s, 60 °C for 30 s and 72 °C for 30 s. Melting curve analysis was performed at the end by cooling to 60 °C, followed by heating to 95 °C at 0.05 °C/s. Automated data analysis was generated with the Hyris data analysis platform. Two technical replicates were performed on the platform for each biological replicate. Three biological replicates were included for each assay.

### Absolute quantification of viral copy numbers through qRT-PCR

*In vitro-*transcribed RNA of the E gene of ZIKV and the 3′-UTR region of DENV2 were used for absolute quantification. Viral total RNA was extracted using TRIzol Reagent (Invitrogen, Thermo Fisher Scientific) from viral cell line cultures. RNA (2 µg) was used for cDNA synthesis with M-MLV Reverse Transcriptase (Promega, Madison, WI, USA). Conventional PCR was used with the primers (Table 1) to amplify a DNA fragment of 168 bp for DENV2 and 145 bp for ZIKV. PCR products were purified using a QIAquick PCR Purification Kit (Qiagen). About 50 ng of cleaned PCR product of either DENV2 or ZIKV was separately cloned with a TOPO-TA Cloning Kit (Invitrogen, Thermo Fisher Scientific) according to the manufacturer’s instructions. The positive clones were screened through colony-PCR, followed by plasmid mini-prep (QIAprep Spin Miniprep Kit, Qiagen) and sequencing confirmation using the same primer sets. The plasmids of the final confirmed-positive clones were purified using the plasmid Maxi-prep Kit (Qiagen). Plasmid DNA was transcribed using HiScribe T7 High Yield RNA Synthesis Kit (New England Biolabs Inc, Ipswich, MA, USA) and the concentration of the RNA was measured with a NanoDrop Spectrophotometer (Thermo Fisher Scientific). Molecular weights were converted to copy numbers using the New England Biolabs Calculator (https://nebiocalculator.neb.com/#!/ssrnaamt). The concentration of RNA was adjusted to 10^10^ copies/µl and serially diluted 10 times for the standard curve on the bCUBE. The standard curve was generated using GoTaq 1-Step RT-qPCR (Promega, Madison, WI, USA).

### Statistical analysis

Graphs were generated using GraphPad Prism Software version 8. The estimation of several diagnostic parameters for leg samples, including sensitivity, specificity, accuracy and positive and negative predictive values was calculated using the web-based software MedCalc Diagnostic Test Evaluation Calculator.

## Results

### Validation of the portable bCUBE qPCR machine to a laboratory-standard qPCR system

**Fig. 1:**
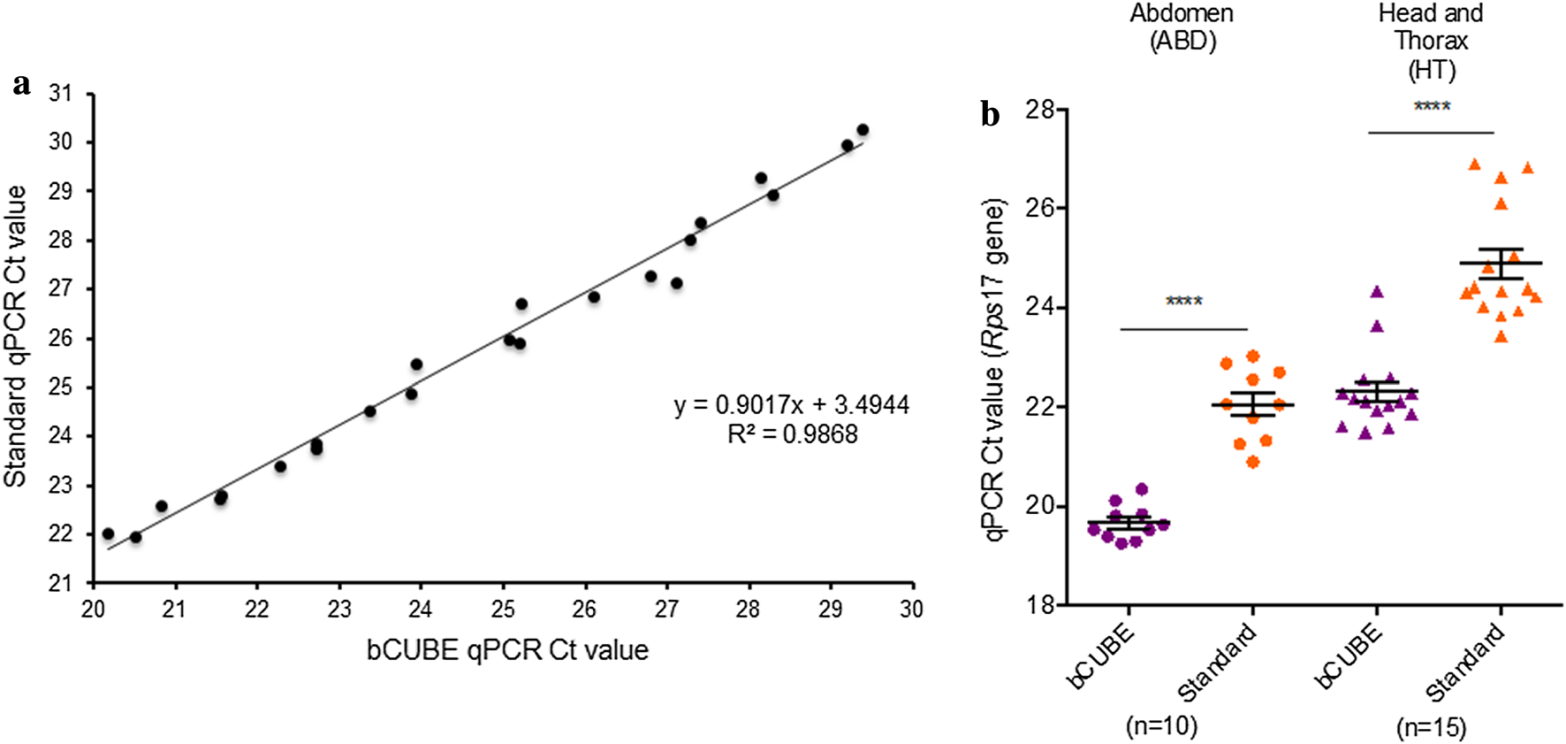
Comparison of *Ae. aegypti Rps*17 gene detection in serially diluted cDNA and individual tissue samples using bCUBE and laboratory standard real-time qPCR. Correlation coeffient was calculated for serially diluted *Ae. aegypti* cDNA using *Rps*17 (**a**)*.* The bCUBE qPCR Ct values are plotted on the x-axis while standard qPCR Ct values are plotted on the y-axis. The housekeeping gene *Rps*17 was amplified using the bCUBE and laboratory standard qPCR systems from the crude mosquito tissue homogenates (**b**). Cycle threshold (Ct) values are plotted of abdomen (circles) and head with thorax (triangles). Statistical significance was determined by paired t-test (****P < 0.0001). *Abbreviations*: *Rps*17, ribosomal protein 17 gene; ABD, abdomen with midgut; HT, head with thorax

The novel bCUBE real-time PCR system was validated by comparing the system to a standard qPCR instrument (StepOnePlus, Applied Biosystems, ABI). We performed the qPCR assays on both machines with serially diluted *Ae. aegypti* cDNA samples with the mosquito housekeeping gene *Rps*17 and same qPCR master mix. The *R*^2^ value of the Ct values from both machines is 0.98678 demonstrating a strong association between bCUBE and standard qPCR (Fig. 1a).

To validate our squash buffer extraction method with bCUBE and laboratory standard, our proof-of-principle studies used *Ae. aegypti* mosquito tissue homogenates (ABD, HT) in squash buffer that were tested with the mosquito housekeeping gene *Rps*17 using both qPCR machines. *Rps*17 gene was detected in all 25 samples by both machines. The cycle threshold (Ct) values for the *Rps*17 gene were detected significantly earlier in the bCUBE system than laboratory standard StepOnePlus, with a mean difference of 2.49 Ct value (paired t-test, *t* = 13.65, *df* = 24, *P*-value < 0.0001) (Fig. 1b).

### bCUBE-based one-step qRT-PCR is sufficiently sensitive for DENV2 and ZIKV detection

We performed sensitivity studies on the bCUBE-based qPCR system based on absolute quantification standard curve analysis. *In vitro-*transcribed cloned fragments of DENV2 and ZIKV were quantitated for RNA copy numbers and serially diluted to establish qPCR standard curves. Based on the standard curves the cut-off Ct value was set to ≤ 30 (approximately 1–10 RNA copy numbers) and ≤ 29 (approximately 1–10 RNA copy number) for ZIKV and DENV2, respectively (Additional file 1: Figure S1). Amplification above these Ct values was attributed to non-specific amplification. Correlation coefficient (*R*^2^) values were 0.99 for DENV2 and 0.98 for ZIKV using standard curve.

### bCUBE-based qRT-PCR is specific for DENV2 and ZIKV detection

The specificity of the ZIKV and DENV2 assays was then determined using a cross-reactivity panel. To address challenges involving primer cross-reactivity with other arboviruses [44, 46] as well as the tendency toward false-positive amplification in negative samples, we evaluated the specificity of the assay against a panel of *Flaviviruses* and one *Alphavirus* using established primers (Table 1). The ZIKV and DENV2 primer pairs showed a high degree of specificity for amplification of their respective virus RNAs (Table 2), since no amplification of other virus RNAs occurred. Furthermore, serially diluted ZIKV and DENV2 virus (10^8^–10^0^) did not cross-react with their primer sets. The DENV2 primer pair did not amplify ZIKV at any RNA viral copy number dilution. ZIKV primer pairs amplified 10^8^ copy number of DENV2 at a Ct of 29.61, however showed a different melting curve peak of 67.3 °C as opposed to that of the positive control at 80.8 °C (Additional file 2: Table S1). This would suggest a false positive amplification of this DENV2 using the ZIKV bCUBE assay. Furthermore, NCBI BLAST did not reveal any similarities with other viruses or potential environmental contaminants. The forward primer for DENV2 was specific for DENV2 isolates, whereas the DENV2 reverse primer showed similarities with Dengue virus 1 and 3 (DENV1-3) isolates. This is in agreement with the origin of the viral primer as it was used for detecting DENV1-3. BLAST searches for ZIKV primer pairs revealed similarities only to ZIKV isolates.

### bCUBE-based qRT-PCR and plaque assay show insignificant differences in detecting infected mosquitoes

**Fig. 2:**
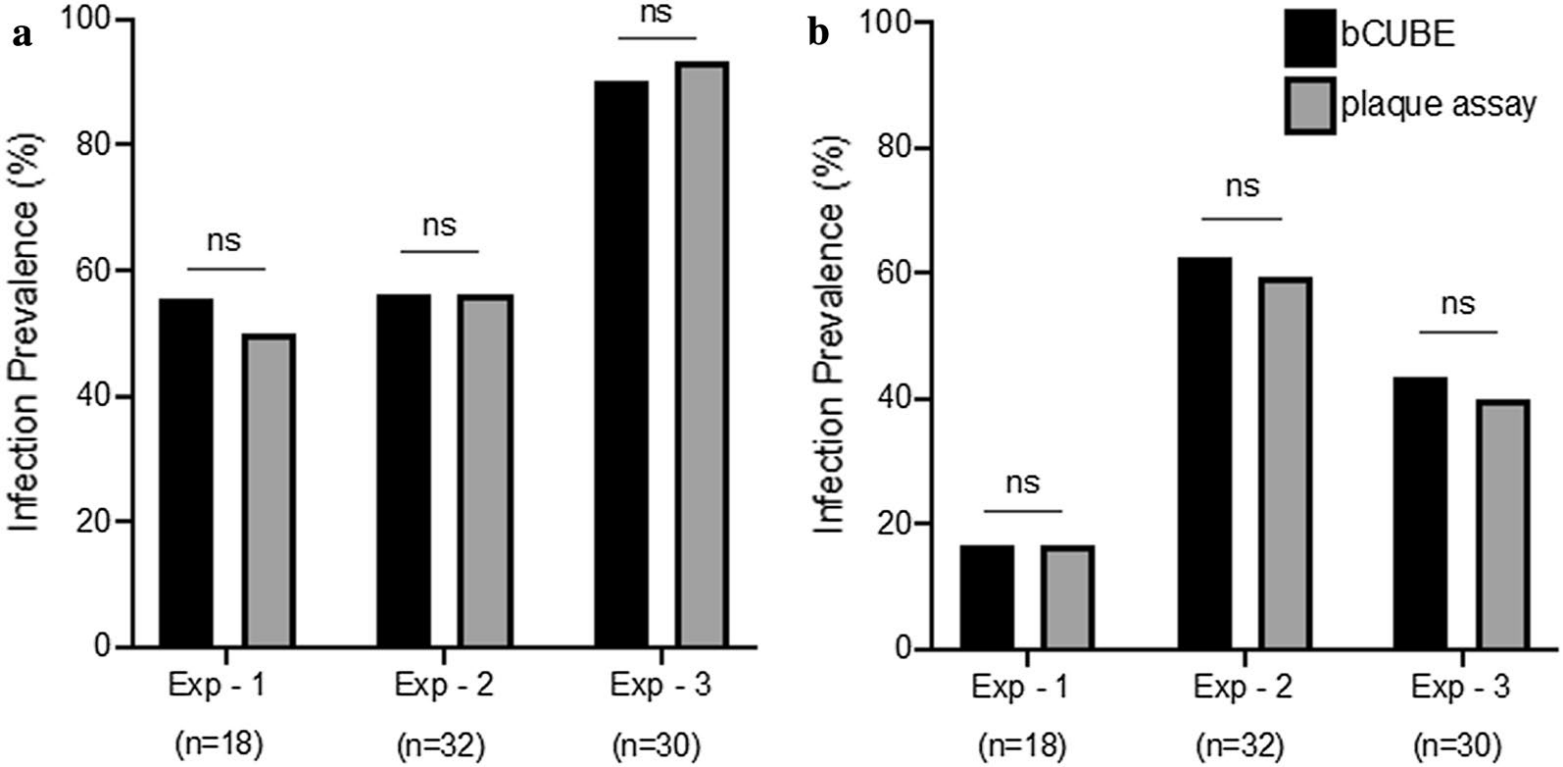
ZIKV and DENV2 infection prevalence in *Ae. aegypti* as detected in the bCUBE *versus* plaque assay. *Aedes aegypti* were infected with Zika (**a**) and dengue (**b**) virus via an artificial blood meal. Each group was split into two groups and analyzed using the bCUBE assay (black bars) and the other half was used for the plaque assay (grey bars). Three experiments (Exp-1, Exp-2, Exp-3) were done for both ZIKV and DENV2 infected individual mosquitoes. No significant difference was detected between the plaque assay and bCUBE qPCR in terms of infection prevalence (% of infected mosquitoes) (ns, not significant; Fisher’s exact test). *Abbreviations*: ZIKV, Zika virus; DENV2, dengue virus serotype 2; Exp, experiment

qRT-PCR based methods are known to detect the genomic RNA of both intact and inactivated viruses [47]. Therefore, the viral genomic RNA copies and infectious viral loads can differ in mosquitoes. We thus sought to compare the infection prevalence (% of infected mosquitoes) determined by bCUBE-based qRT-PCR assay and plaque assays as opposed to titer comparisons. Three experiments were performed with *Ae. aegypti* infected with ZIKV (*n* = 160) and DENV2 (*n* = 160). Each cohort of infected mosquitoes was separated into two halves. One half of the cohort was collected individually in squash buffer (for bCUBE qPCR) and the other half was collected in complete DMEM medium (for plaque assay) to compare infectious prevalence. In each of the three experiments, insignificant differences were shown for both ZIKV and DENV2 between the two methods (Fig. 2a, b). bCUBE qPCR detects more infected mosquitoes than plaque assays at insignificant levels. These results suggest that bCUBE qRT-PCR analysis is as reliable as laboratory-based plaque assay in detecting ZIKV and DENV2 infected mosquitoes. No correlation was observed between Ct values from bCUBE-based qPCR assays and viral titers from plaque assays (data not shown).

### ZIKV and DENV2 RNA can be detected and quantified in individual mosquito samples by bCUBE assay

**Fig. 3:**
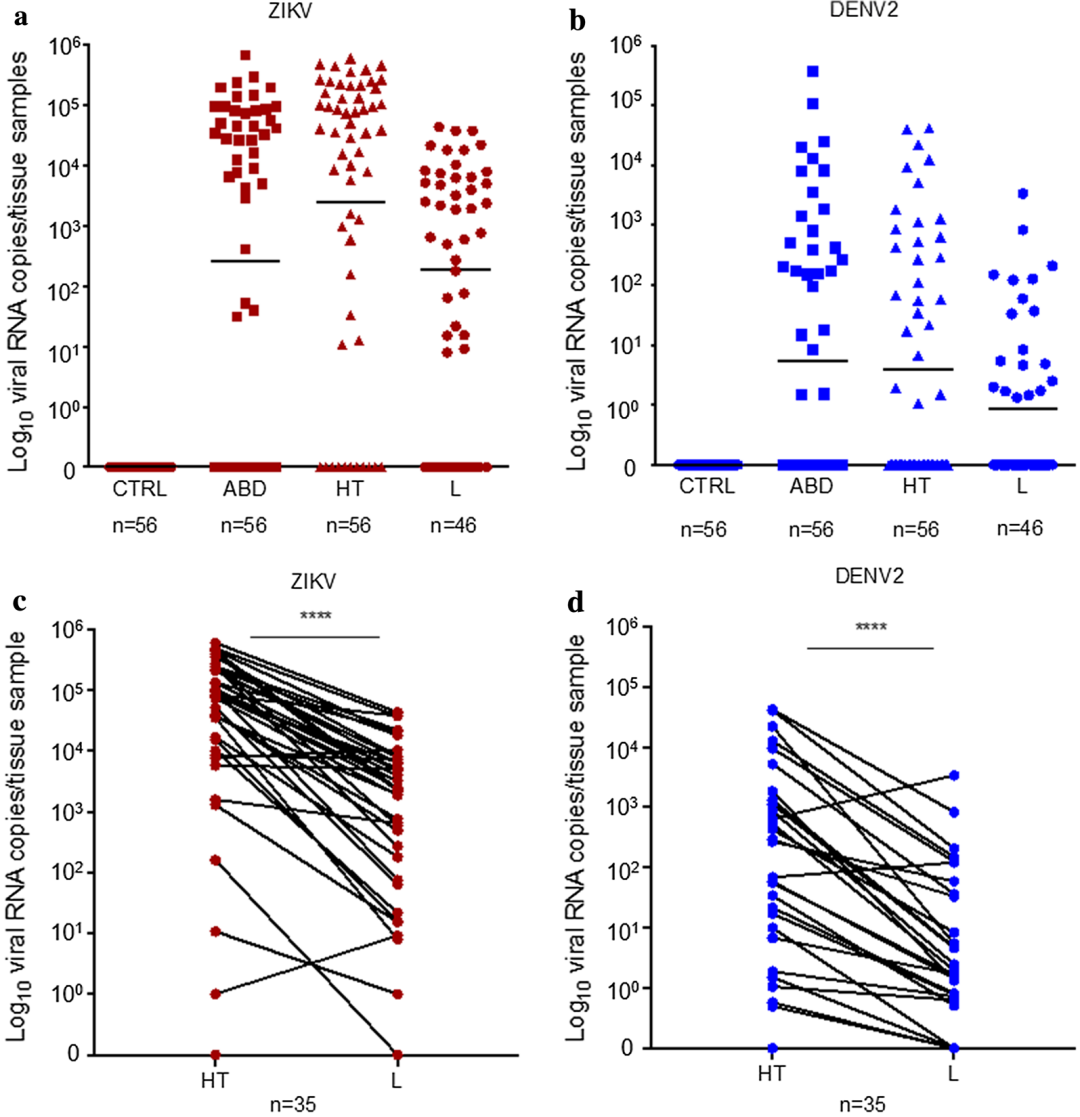
Viral RNA concentrations of individual *Ae. aegypti* tissues collected at various time points. Individual *Ae. aegypti* that were infected with Zika (**a**) and dengue virus (**b**) and collected at 7 or 14 days post-infectious blood meal. Squares represent abdomens with midgut samples (ABD) collected on day 7 post-infectious blood meal to measure the viral loads in the midgut stage. Triangles represent head with thorax samples (HT) and circles represent corresponding leg (L) samples were collected on day 14 to evaluate the disseminated viral loads. Uninfected *Ae. aegypti* are included as negative controls. The vertical axis represents RNA viral genome copy number per tissue sample derived from absolute standard curve. Infected *Ae. aegypti* (*n* = 46) were collected and identical samples of head with thorax (HT) and legs (L) were amplified for Zika virus (**c**) and dengue virus serotype 2 (**d**) using bCUBE-based qRT-PCR. Each circle represents individual mosquito tissue amplified by bCUBE qRT-PCR. The vertical axis represents RNA viral genome copy number derived from standard curve. Infection status was compared (****P < 0.0001; paired two-tailed t-test). *Abbreviations*: ABD, abdomen with midgut; HT, head with thorax

*Aedes aegypti* mosquitoes were experimentally infected with ZIKV (*n* = 112) and DENV2 (*n* = 112) and the squash buffer method was used to extract total RNA of the tissues in a single infected mosquito. Uninfected *Ae. aegypti* (*n* = 56) were used as negative controls. Both ZIKV and DENV2 viral RNA were detected using bCUBE qPCR with a single infected mosquito tissue. Viral RNA could be quantitated in ABD tissues at day 7 (indicating the midgut-stage infection) and HT tissues at day 14 (indicating disseminated viral infection). No uninfected negative controls were amplified using the ZIKV or DENV2 qPCR assay (Fig. 3a, b).

Next, we investigated whether bCUBE qPCR detection method is sensitive for detection of disseminated virus in the leg samples rather than HT tissues (Fig. 3a, b). Forty-six leg samples were collected in parallel with the collection of HT samples assayed above. Paired t-test comparison of the Ct values of HT samples *versus* the leg samples has shown significant differences in DENV2 (paired t-test, *t* = 6.099, *df* = 45, *P* < 0.0001) and ZIKV (paired t-test, *t* = 7.972, *df* = 45, *P* < 0.0001) (Fig. 3c, d). For the leg samples, six false negative sample results were obtained for DENV2 and two false negative results were obtained for ZIKV. However, this is likely due to low amount of ZIKV and DENV2 RNA present in leg samples and therefore below the detection threshold. One false positive was detected from a ZIKV infected leg sample. These results suggest using caution when processing leg samples for DENV2 and ZIKV detection. No amplification occurred in uninfected negative controls.

The sensitivity of the bCUBE qPCR assay for the leg samples was 76% (DENV2, 95% CI: 54.87–90.64%) and 94.59% (ZIKV, 95% CI: 81.81–99.34%). The specificity of the bCUBE qPCR assay using leg samples was 100% (DENV2, 95% CI: 83.89–100%) and 88.89% (ZIKV, 95% CI: 51.75–99.72%). The overall accuracy of the bCUBE qPCR for leg samples was 93.48% for ZIKV (95% CI: 82.10–98.63%) and 86.96% for DENV2 (95% CI: 73.75–95.06%) (Fig. 3c, d).

### bCUBE-based qRT-PCR detects ZIKV and DENV2 viral RNA in pooled *Ae. aegypti* samples

Surveillance programmes monitoring ZIKV and DENV2 transmission will typically assay the presence of virus in pooled samples ranging from 5 to 50 mosquitoes [48]. We spiked pools of 4, 9, 14 and 19 uninfected *Aedes* with individual ZIKV- or DENV2-infected mosquitoes to assess the potential of using this assay with pooled samples in the field. The infection status of one single infected mosquito was confirmed by viral RNA extraction using squash buffer followed by bCUBE-based qRT-PCR. As shown in Table 3, ZIKV could be detected in all pooled samples with various viral load input (approximately 47–390,085 copies). DENV2 could be detected in all three pools of 5-, 10- and 15-pools. DENV2 viral RNA could not be detected in one of the 20-pools with low viral load input (approximately 47 copies) of a single infected mosquito, whereas in one of the 10-pools the input at approximately 49 copies showed positive detection, suggesting bCUBE qPCR assay is sensitive to detect viral infection in the smaller mosquito pools. Significant differences exist between individual mosquito titer and the pooled titer for ZIKV (Wilcoxon signed rank test, *P* < 0.0005) and DENV2 (Wilcoxon signed rank test, *P* < 0.0005) suggesting that larger quantities of mosquitoes reduce the ability to detect virus.

**Table 3:** Detection of viral RNA in pooled *Aedes aegypti* samples by bCUBE qRT-PCR

	Pool size											
	5			10			15			20		
	Titer of individual mosquito^a^	Titer of pool^a^	No. of positives	Titer of individual mosquito^a^	Titer of pool^a^	No. of positives	Titer of individual mosquito^a^	Titer of pool^a^	No. of positives	Titer of individual mosquito^a^	Titer of pool^a^	No. of positives
ZIKV	47	15	3/3	52	9	3/3	6666	45	3/3	58	8	3/3
	4181	88		17,095	68		10,721	239		27,257	641	
	19,629	1808		383,403	1989		193,745	4480		390,085	10,629	
DENV2	760	34	3/3	49	2	3/3	89	1	3/3	47	UDL	2/3
	1471	17		618	48		1846	3		12,844	54	
	152,817	1682		21,077	14		27,848	157		59,144	279	

^a^RNA copy number determined by reverse transcriptase-polymerase chain reaction standard curve

*Notes*: Pooled samples were prepared by combining individual ZIKV- and DENV2-infected mosquitoes with pools of uninfected mosquitoes. Pools of 4, 9, 14, or 19 uninfected mosquitoes plus one infected mosquito were evaluated by bCUBE qRT-PCR. Table lists number of positive pooled samples detected by bCUBE qPCR and the titer of individually infected mosquitoes and corresponding pooled samples

*Abbreviation*: UDL, under detection limit

## Discussion

Real-time qPCR has long been considered the gold standard for viral detection; however, its potential application in the field has been limited by the requirement for bulky equipment and limited ability for on-site sample preparation. This is particularly important in the field of mosquito-transmitted diseases, such as dengue and Zika, where viral surveillance of mosquitoes is regularly conducted in disease-endemic regions that may lack nearby laboratory facilities to run qPCR diagnostics. Here, we explored the use of a novel, portable qPCR device, bCUBE as a platform for detecting arboviruses in *Ae. aegypti*, with a view towards future field-deployment.

We validated the bCUBE by comparing its performance against a laboratory qPCR standard and found it is capable of performing real-time qPCR while overcoming the barriers presented by the need for bulky laboratory standard equipment. We were able to detect ZIKV and DENV2 RNA in bCUBE with equal sensitivity to plaque assay. Because qPCR is capable of detecting low RNA copy numbers in individual samples, it is important to evaluate the assay’s potential for detecting infectious viral RNA rather than viral RNA that is not replicating [47, 49]. No significant differences were detected between the bCUBE and plaque assay for three experimental studies, suggesting that the bCUBE assay is able to detect viral nucleic acid with equal sensitivity as plaque assay. However, it is relevant to note that the presence of viral RNA does not always indicate infectious virus. Viral isolation from cell culture has been the traditional method for detection of infectious virus, this method requires time, expertise, equipment, and low sensitivity [50]. Furthermore, we could detect both viral samples in pools of 5, 10 and 15 mosquitoes highlighting the bCUBE’s potential for field application. The assay was specific for both ZIKV and DENV2, as it did not show cross-activity to other frequently occurring viruses.

### A low-cost qPCR assay suitable for arboviral detection in the field

Our primary goal was to develop and optimize a low-cost qPCR-based assay for arbovirus detection in mosquito samples using the bCUBE that was compatible with use in field conditions. Arguably, the start-up price for a qPCR machine is a major concern for surveillance programmes. Hyris bCUBE (Hyris, London, UK) is 50–80% more cost effective in start-up fees than many standard qPCR machines on the market, including the CFX96, StepOnePlus and LightCycler. Furthermore, the maximum power consumption is 60 W while it maintains a 20 W average during PCR cycling. For comparison, standard qPCR machines typically use 850 W. This allows for the use of an external 1.5-kg battery pack with a four-hour run time further highlighting the capacity for this qPCR to be run remotely. However, as the market begins to expand, there is a rise in portable qPCR machines under development and being released for laboratory validation.

There are multiple challenges associated with performing qPCR assays in the field, particularly related to the stability and cost of reagents. To that end, we were mindful of the need to limit the cost per sample and sought to utilize reagents that were easy to use, highly stable and low cost. We utilized an RNA extraction protocol based on squash buffer and proteinase K, which are both stable at room temperature and allow for a crude extraction of mosquito DNA/RNA, at an estimated cost of $0.60–0.70 per reaction (USD). This extraction method was validated with our laboratory standard and bCUBE where individual mosquito tissues were detected with *Rps*17 molecular markers using both machines. Furthermore, it preserved virus for sensitive detection when compared to plaque assay making it an ideal extraction method for field use. However, RNA extraction using these reagents can be less clean that what can be achieved through other protocols, and can result in issues during downstream applications [51]. It is important to note that current bCUBE cartridges are restricted to 16-wells thereby limiting the number of samples run during each experiment. The costs of cartridges are currently priced at an estimated cost of $0.20 per reaction (USD) which is nearly four times the cost of well-plates for standard qPCR running larger reactions. The size of the cartridge limits the potential for performing more reactions at a faster turnaround time. However, Hyris plans to address these concerns by the release of a new cartridge format that includes 36-wells allowing for increased testing throughput (L. Colombo, personal communication, July 6, 2020). Although this cartridge type still has limitations of only running 36 reactions, it does allow for processing more than double the reactions as before.

As an alternative to hydrolysis probe-based qPCR kits, which can be quite expensive, we used DNA dye binding qPCR commercial kits. Because the bCUBE has only two detection channels, we were limited by our analyses for any probe-based qPCR kits and found that DNA dye binding qPCR kits were suitable for our aims. When compared to plaque assay, our ZIKV and DENV2 detection was equally sensitive. The GoTaq 1-step qPCR kit and primers need to be kept on ice, which is a complication for field-deployment faced by many detection systems [52]. However, it is an issue that could be overcome through the proposed release of bCUBE-compatible pre-filled cartridges with lyophilized PCR reagents (L. Colombo, personal communication, July 6, 2020). Similarly, positive controls must also be kept on ice under field conditions, and while this is an issue common to all field-based qPCR applications, it is a limitation that could hopefully be overcome in the future.

### Technical considerations for field deployment

There are multiple issues associated with the primers used in qPCR diagnostics that could arise during arbovirus detection in the field. To address challenges involving primer cross-reactivity with other arboviruses [44, 46], as well as the tendency toward false-positive amplification in negative samples, we tested the assay against a panel of frequently co-circulating arboviruses. Our results confirmed the assay’s specificity for DENV2 and ZIKV RNA, and the potential for applying these primer pairs in field conditions. Although these are promising data, they still need to be tested for accuracy in the face of genetic change to arboviral genomes that might be observed in the field, where there is a high frequency of viral mutation. This tendency highlights the importance of vigilantly monitoring the viral strains that are co-circulating in a region, in conjunction with viral detection in mosquitoes, in order to accurately and specifically detect viral RNA in mosquitoes. This information can be used to inform the selection of appropriate primers for a given region, over time.

The limit of detection identifies the lowest amount of RNA copy number that can be positively detected by qPCR in a given sample [17]. In our study, we used absolute standard curves to determine cut-off values for detecting ZIKV and DENV2 to understand the lowest copy number that can be detected in the laboratory and under field conditions. Amplification at later cycles demonstrated false positive amplification as a result of background noise. Borderline positive PCR results with high Ct values may pose a challenge for field deployment because they may be due to false positives, incredibly low viral load, or cross-contamination. However, these false positives can also be verified using melting curve analysis. For future studies in the field, these high Ct value samples can be sequenced for further analysis to confirm infection. Setting a cut-off value on the bCUBE software settings, allowed for true positive identification of viral RNA from infected mosquito samples and limits training for personnel when applying this technology to the field in the future. It is critical to perform absolute quantification analyses and determine end-point limitation values during optimization to avoid amplification of false positive samples.

Whole mosquitoes will typically be used for several analyses including infection status, microbiome analysis, gene expression studies, identification, or insecticide resistance detection. In addition, the analysis of leg samples permits the mosquito to stay alive, if necessary, in a laboratory setting. Developing an assay for the detection of virus in leg was therefore relevant and we evaluated the use of leg samples for viral detection. Our investigation demonstrated both false negative and false positive results for ZIKV and DENV2. This can be attributed to a variety of factors including low viral presence, contamination, presence of inhibitors, or viral RNA degradation. Taking this into account, using legs for detecting virus should be proceeded with caution using this methodology. However, mosquito surveillance agencies typically evaluate arboviral infection status in pooled samples as opposed to assaying individual mosquitoes or legs.

The detection of a positively-infected mosquito in the general population is low; therefore, surveillance programmes will pool samples to increase the probability for detecting virus and limiting the cost and time of sample processing [48]. Data shown in our study suggest that the squash extraction methodology coupled with bCUBE qPCR will be capable of detecting ZIKV and DENV2 in the field. ZIKV RNA was detected in pools of 5, 10, 15 and 20 with uninfected mosquitoes in three biological replicates. In our study, we used various viral copy numbers, including low to high, to combine with pools as opposed to only high titers. This would mimic similar conditions in the field with various infected mosquitoes in collection pools. DENV2 RNA was not detected in one of the 20-mosquito-pools. The inability to detect virus in the 20-pool could be attributed to low viral copy number for the infected mosquito (47 copies), suggesting possible limitations for DENV2 detection in larger pools with a low infected mosquito. However, overall this highlights the bCUBE platform’s potential for pool testing in the field.

To expand the versatility of the bCUBE for vector surveillance, we performed preliminary experiments aimed at detecting *Wolbachia* in *Ae. aegypti. Wolbachia pipientis* is an endosymbiotic bacterium that is passed from mother to offspring and has been shown to suppress dengue and Zika virus transmission in *Aedes* mosquitoes [53, 54]. Therefore, it is increasingly being used as a method for limiting arboviral transmission in dengue and Zika virus-endemic countries. Field-release trials require continual monitoring of *Wolbachia*-infected *Ae. aegypti* in mosquito populations [55–57]. Real-time qPCR has been used as the primary method for detecting *Wolbachia* genetic material in mosquitoes [58]. Our investigation revealed successful amplification of *Wolbachia* in adult *Ae. aegypti* (*n* = 35) using established primers (Additional file 3: Figure S2). These results indicate the bCUBE’s potential beyond arboviral detection. In parallel with arboviral detection, the bCUBE may be used for *Plasmodium falciparum* detection in *Anopheles* species, as well as monitoring the increasing rate of insecticide resistance in the future. The bCUBE can provide a powerful multi-panel platform for monitoring different mosquito species, their diseases, and insecticide resistance.

## Conclusions

We have developed a simple, low-cost and highly sensitive qPCR-based assay for the detection and quantitation of DENV2 and ZIKV in individual or pooled mosquitoes using the portable qPCR Hyris bCUBE platform. Our assay allows for sensitive and accurate DENV2 and ZIKV detection and is a highly promising potential tool that could be utilized by mosquito surveillance programmes in countries facing arboviral outbreaks. The stability and low cost of the extraction technique used coupled with a portable qPCR make it ideal for field deployment. This platform is highly versatile, with our data revealing rapid and accurate detection of the dengue-blocking endosymbiotic bacterium *Wolbachia* in *Ae. aegypti* and suggesting broad applications for mosquito-transmitted disease. By overcoming the challenges of costs associated with reagents and equipment, the bCUBE qPCR platform offers a highly promising and potentially field-deployable laboratory resource that could prove to be particularly valuable for mosquito and arboviral surveillance agencies in remote regions.

## Supplementary information

**Additional file 1: Figure S1.** Cycle threshold (Ct) standard curves for DENV2 and ZIKV generated using GoTaq 1-Step RT-qPCR (Promega) and bCUBE qPCR. Absolute quantification was based on standard curve analyses using cloned fragments from the DENV2 and ZIKV stocks. Viral RNA was adjusted to 10^10^ copies and serially diluted 10 times for qRT-PCR. Cycle threshold (Ct) values are plotted against log_10_ of RNA copy numbers (RNA copies/µl).

**Additional file 2: Table S1.** Cycle threshold (Ct) and melting curve peak values for cross reactivity panel. DENV2 and ZIKV were serially diluted (1 × 10^8^–1 × 10^0^) for qRT-PCR and amplified with the opposite primer pairs. For instance, DENV2 was amplified with ZIKV primer pairs and ZIKV was amplified with DENV2 primer pairs. Viral RNA copy number is listed with corresponding amplification Ct and melting curve peak values.

**Additional file 3: Figure S2.** Cycle threshold (Ct) values of *Wolbachia*-infected *Ae. aegypti.* Previously developed primers were used for SYBR green qPCR on the Hyris bCUBE platform to amplify *Wolbachia-*infected *Ae. aegypti* (*n* = 35). Ct values ranged from 18.92 to 26.48.

## Data Availability

The datasets used and analyzed for this study are available from the corresponding author upon reasonable request.

## Abbreviations

ZIKV: Zika virus
DENV2: dengue virus serotype 2
qPCR: quantitative real-time polymerase chain reaction
qRT-PCR: quantitative real-time reverse transcription polymerase chain reaction
RNA: ribonucleic acid
gDNA: genomic deoxyribonucleic acid
ABD: abdomen with midgut
HT: head with thorax
L: leg
BEI Resources: Biodefense and Emerging Infections Research Resources Repository
CHIKV: chikungunya virus
JEV: Japanese encephalitis virus
WNV: West Nile virus
YFV: yellow fever virus
PIBM: post-infectious blood meal
