# Supplementary figures and images for: Field-deployable molecular diagnostic platform for arbovirus detection in Aedes aegypti
Source: Parasit Vectors. 2020 Sep 24;13:489. doi: 10.1186/s13071-020-04357-y (PMC7513541; doi:10.1186/s13071-020-04357-y)

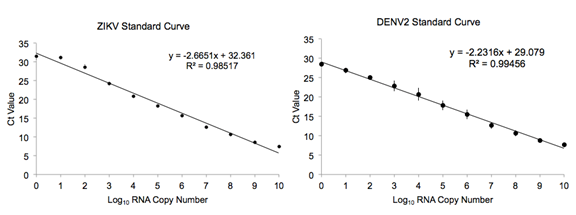

Supplement: Supplementary file 1 — Additional file 1: Figure S1. Cycle threshold (Ct) standard curves for DENV2 and ZIKV generated using GoTaq 1-Step RT-qPCR (Promega) and bCUBE qPCR. Absolute quantification was based on standard curve analyses using cloned fragments from the DENV2 and ZIKV stocks. Viral RNA was adjusted to 1010 copies and serially diluted 10 times for qRT-PCR. Cycle threshold (Ct) values are plotted against log10 of RNA copy numbers (RNA copies/µl). [file 13071_2020_4357_MOESM1_ESM.tif]

**Additional file 2: Table S1.**


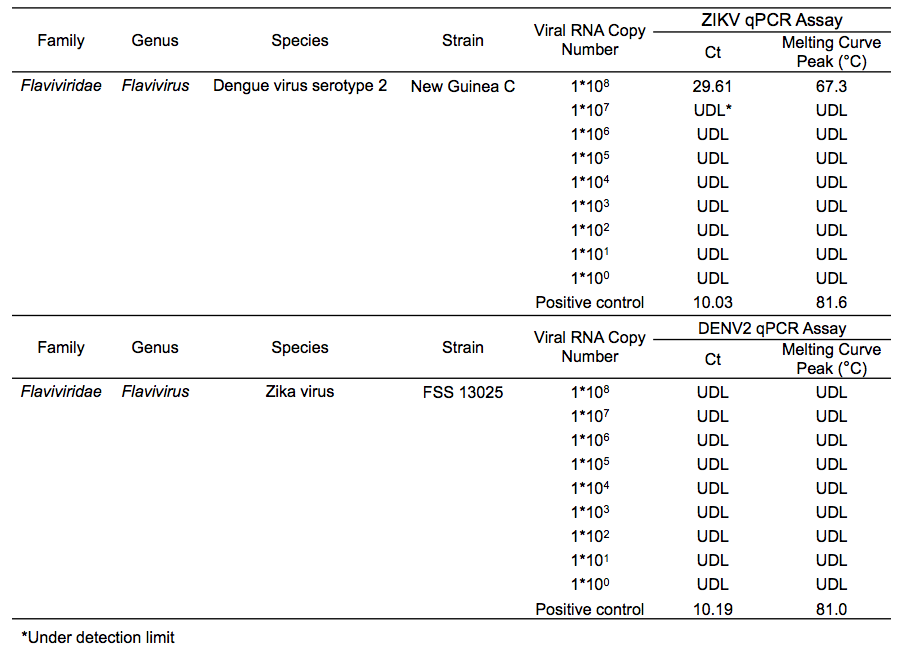

Supplement: Supplementary file 2 — Additional file 2: Table S1. Cycle threshold (Ct) and melting curve peak values for cross reactivity panel. DENV2 and ZIKV were serially diluted (1 × 10^8^–1 × 100) for qRT-PCR and amplified with the opposite primer pairs. For instance, DENV2 was amplified with ZIKV primer pairs and ZIKV was amplified with DENV2 primer pairs. Viral RNA copy number is listed with corresponding amplification Ct and melting curve peak values. [file 13071_2020_4357_MOESM2_ESM.docx]

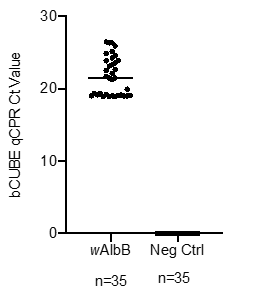

Supplement: Supplementary file 3 — Additional file 3: Figure S2. Cycle threshold (Ct) values of Wolbachia-infected Ae. aegypti. Previously developed primers were used for SYBR green qPCR on the Hyris bCUBE platform to amplify Wolbachia-infected Ae. aegypti (n = 35). Ct values ranged from 18.92 to 26.48. [file 13071_2020_4357_MOESM3_ESM.tif]
